# Debate: Social media in children and young people – time for a ban? It is time to take a precautionary approach. Why health professionals are calling for a ban on social media for under‐16s

**DOI:** 10.1111/camh.70037

**Published:** 2025-09-23

**Authors:** Arabella Skinner, Rebecca Foljambe

**Affiliations:** ^1^ Health Professionals for Safer Screens London UK; ^2^ GP Partner, Sleights & Sandsend Medical Practice York UK

**Keywords:** Age verification of 16 years, safeguarding children, public health crisis

## Abstract

As frontline health professionals working across paediatrics, psychiatry, psychology and general practice in the United Kingdom, we are witnessing an alarming and growing mental health crisis among children and adolescents, which we believe is exacerbated by social media use. Drawing upon clinical experience and supported by a growing body of research, we present evidence that social media contributes to a spectrum of adverse mental health outcomes, including anxiety, depression, eating disorders, body dysmorphia, self‐harm and suicidality. Particularly vulnerable populations, including neurodivergent children and those facing socioeconomic disadvantage, are disproportionately affected. Despite academic suggestions of some benefits, our real‐world experience of the preponderance of clinical cases indicates an urgent need for preventive action. We argue that current regulatory frameworks are insufficient and propose a precautionary public health approach: an immediate statutory ban on social media use for all children under 16, placing the burden of proof on technology companies to demonstrate safety before allowing access. We further advocate for the strengthening of age verification systems, public health campaigns, parental guidance interventions and routine clinical screening for problematic social media use. This paper reflects the collective voice of our health professionals on the frontline of child and adolescent care, calling for decisive policy action to address a preventable and escalating threat to youth mental health.

At Health Professionals for Safer Screens, a collective of UK clinicians, we have reached a critical conclusion: social media use for children under 16 must be banned. Social media is not harmless; it can contribute to or provoke worsening symptoms of anxiety, depression, disordered thinking around body image, eating disorders and substance abuse. We are witnessing firsthand in our clinics the ability of social media to drive down children's self‐esteem, fuel and perpetuate ideas of self‐harm and at worst, directly contribute to a child's death by suicide, either by leading them into the darkest rabbit holes of despair on social media or provoking them into an impulsive act to complete a fatal craze or challenge.

As health professionals, we see the impact of our young people's all‐time low mental health and one in four children and young people use their smartphones in a way that is consistent with a behavioural addiction (Sohn, Rees, Wildridge, Kalk, & Carter, 2019). While some research indicates that social media may occasionally benefit children, this does not outweigh the significant harm they experience on these platforms. There is no substantial evidence supporting the safety or benefits of social media for children. Our clinical frontline professionals have seen enough in their daily clinics to conclude that social media is not a proven healthy or safe place for children.

We are calling for a precautionary approach: instead of waiting for definitive evidence that social media is dangerous, the burden of proof should lie with definitive evidence of its safety. We apply the precautionary principle every day in clinical practice. For example, when treating children with tics or other behavioural issues or prescribing certain off‐licence medications, we often do so without comprehensive guidance or conclusively researched procedures. We do so with care and caution, based on our experience and current knowledge. We need to apply it now to social media and enact legislation to force social media companies to take full responsibility for age‐verifying their users, with immediate consequences when they are found to be in breach. It is the very least we owe our children.

## A frontline view of the crisis

Every day, clinicians in paediatrics, psychiatry, psychology and general practice are seeing children and adolescents whose mental health (and physical health) has been directly affected by social media. Whether it is a 12‐year‐old struggling with obsessive‐compulsive behaviours, a 14‐year‐old presenting with severe anxiety or depression, or an 11‐year‐old girl with an eating disorder, the pattern is clear. Social media is not a benign part of modern life; it is, in our experience, a potentially toxic and coercive space that children and young teens are more vulnerable to than at any other stage in their lives.

Our clinical practice has shown that many of the children we see have encountered violent, disturbing or even extreme content on social media. Research backs this up: half of UK children who have witnessed online violence encountered it through someone else's feed and 25% were exposed to it through platform promotion (Youth Endowment Fund, 2024). In our clinics, we regularly hear reports of children being led down a rabbit hole of harmful content after interacting with seemingly innocent material, such as a beauty tutorial or a fitness influencer.

Our clinical experience of children from vulnerable backgrounds being more adversely impacted by exposure to this content and other harmful online spaces and social media feeds is mirrored in reports. Social media harms do not affect all children equally. In short, offline vulnerability, including neurodiversity, poverty and minority groups, extends to online life and digital risk is magnified in children already facing social or economic disadvantage (El Asam & Katz, 2018).

We have seen these interactions translate into real‐world consequences – from escalating mental health issues like anxiety and depression to body dissatisfaction and eating disorders, and in practice, social media exacerbates these problems. A global review of 50 studies found a strong correlation between social media use and body dissatisfaction, particularly among girls (Dane & Bhatia, 2023). We have seen this in our patients – children developing unhealthy relationships with food and body image – echoing research that links increased screen time and social media use to more eating disorder symptoms in early adolescence (Chu et al., 2024).

Our collective clinical experience is not an isolated observation. Problematic Social Media Use (PSMU) has significant, adverse mental health consequences. Currently, around 11% of adolescents worldwide show addiction‐like symptoms in their use of social media, and this number is steadily rising. Our colleagues in paediatrics, psychiatry and psychology are seeing more young people, particularly girls, who are exhibiting signs of PSMU. In England alone, 20% of 11‐year‐old girls now fall into this category (Boniel‐Nissim et al., 2024). The evidence tells us that children and teens with higher social media use experience heightened feelings of anxiety, depression and attention issues (Nagata, Lee, Hur, & Baker, 2025).

## The public health crisis in plain sight

As health professionals, we see the full scale of the damage. Social media is not just causing a few isolated problems – it is contributing to a widespread public health crisis in our youth. We are seeing spikes in anxiety, depression, eating disorders, substance abuse and self‐harm, all of which are exacerbated by social media use. In our clinics, we observe the significant impact of social media on children's mental health. Most parents believe the risks outweigh the benefits of their children using these platforms. A quarter of 8‐ to 17‐year‐olds using social media feel constant pressure to be popular, with this sentiment rising among 10 to 12‐year‐olds (28% this year vs. 21% last year). The most common way in which children had experienced someone being nasty or hurtful to them was through social media (16%) – as likely as face‐to‐face bullying (15%) – and this was more common among teens in this group than their younger counterparts (20% of 13–17s compared to 11% of 8–12s said they had been bullied via social media) (Ofcom, 2025).

Research from the Global Mind Project indicates that children are spending more time on social media, with earlier exposure leading to severe long‐term consequences. Receiving a smartphone before age 13 is linked to poorer mental health in young adulthood, including suicidal thoughts, emotional instability and reduced self‐worth. Early social media access accounts for 40% of this impact (Thiagarajan, Newson, & Swaminathan, 2025). Studies show that early access to smartphones and social media is linked to increased rates of depression, self‐harm and suicidal thoughts (Nagata et al., 2025). With 27% of UK 3‐ to 4‐year‐olds having their own smartphones and a quarter of those having their own social media profiles (Ofcom, 2025), the time to act is now.

## The current strategy is not working

Across the globe, the answer to the harms of social media for children has almost always been for more digital literacy. The evidence of mounting child mental health admissions shows that this policy alone is inadequate.

Young people themselves recognise the impact of social media on their generation. Campaign groups led by young people, such as Reel It In, are speaking out about the impact social media has had on them. A poll of UK 16‐ to 17‐year‐olds found that one‐third believe the age limit for social media accounts should be 14–15, while 31% favour 16‐ to 17‐year‐olds. Only 4% said there should be no limit (Wheeler, 2025).

It is time for governments to take notice.

## Our call to action: Banning social media for under‐16s

We are calling for the immediate implementation of a statutory ban on social media for all children under the age of 16. In addition, we urge:

1. A public health campaign to raise awareness about the risks of social media for children, led by government health departments.
2. Parental education on creating healthy digital habits at home, including setting screen time limits, establishing phone‐free zones and limiting social media exposure.
3. Regular screening for social media use during all health consultations with children and adolescents to identify potential risks early.

## Conclusion

Evidence from clinical practice and research indicates that social media significantly harms the mental health of children and adolescents. Health professionals have witnessed the devastating effects of unregulated social media, including increased anxiety, depression, eating disorders, self‐harm and suicide. While some studies suggest limited benefits, they do not, in our experience, outweigh the widespread harms we see in clinics.

In light of the lack of conclusive evidence of safety, we advocate for a precautionary approach that prioritises children's well‐being over the interests of technology companies. This includes a statutory ban on social media for children under 16 and strict age verification measures for platforms.

This issue is a public health emergency. As clinicians, we must apply our intervention principles to children's digital environments. It is time for decisive action from policymakers, educators and society, as the cost of inaction is too high for our children.

## Conflict of interest statement

The author has no conflicts of interest to disclose.

## Ethics statement

No ethical approval was required for this debate article.

## Funding information

No funders declared.

## Data Availability

Data sharing is not applicable to this article as no new data were created or analysed.
